# Many Stayers, Few Movers: Seasonal and Sex‐Based Movement Patterns in an Endangered Forest‐Dwelling Salamander

**DOI:** 10.1002/ece3.73900

**Published:** 2026-07-07

**Authors:** Antonio Romano, Andrea Costa, Marco Basile, Giacomo Rosa, Marco Salvatori

**Affiliations:** ^1^ Consiglio Nazionale Delle Ricerche ‐ Istituto per la BioEconomia Roma Italy; ^2^ R.A.M.O.N.E.S – Reptile and Amphibian Monitoring and Ecological Studies Latina Italy; ^3^ Department of Earth, Environment and Life Sciences (DISTAV) University of Genova Genova Italy; ^4^ Swiss Federal Research Institute WSL Birmensdorf Switzerland; ^5^ Research and Museum Collection Office, Conservation Biology Unit MUSE ‐ Science Museum Trento Italy; ^6^ Department of Biology University of Florence Sesto Fiorentino Italy

**Keywords:** amphibian conservation, home range, minimum enclosing circle, *Salamandrina*, SCR modelling, spatial ecology

## Abstract

Understanding movement patterns of low‐mobility forest amphibians is challenging because they are often highly localised, and rare displacement events may disproportionately influence space use, connectivity and population dynamics. We investigated individual and population‐level spatial ecology in the forest specialist *Salamandrina perspicillata* , assessing spatial displacements, movement rates, home ranges and population density across sexes and seasons. We analysed multi‐session capture‐recapture data collected in a temperate forest in central Italy. Spatial displacement was described using cumulative movement distance and maximum step length. Individual space use was estimated using Minimum Enclosing Circles (MEC), while population‐level density and movement scale were assessed through spatial capture‐recapture (SCR) models. Movement rate showed seasonal variation, with significantly higher values during the first autumn compared to spring and second autumn, indicating temporally constrained movement intensity. Distance‐based metrics and MEC home ranges exhibited strongly right‐skewed distributions, with a high proportion of individuals showing no detectable displacement and most mobile individuals remaining within very small spatial extents (median MEC home range: males = 160.22 m^2^; females = 145.30 m^2^). A few individuals accounted for relatively large movements and space use, generating long right tails. Females showed greater inter‐individual variability and a larger movement scale (σ) in SCR models, whereas cumulative distances and home ranges did not differ consistently between sexes after excluding non‐moving individuals. No significant relationships were detected between body size and any space‐use metric. Population density showed marked variation between seasons (between 345 and 728 individuals/ha for males and between 171 and 690 for females), with animals concentrating close to the breeding stream during spring and being more spread out during autumn. Our results unravel a movement system characterised by many highly sedentary individuals and few relatively mobile ones (many stayers, few movers). Integrating multiple techniques, we provided a multi‐scale framework to characterise space use in low‐mobility species that yielded important insights for connectivity and conservation in forest‐dwelling amphibians.

## Graphical abstract

We investigated individual and population‐level spatial ecology in the forest specialist *Salamandrina perspicillata* , assessing spatial displacements, movement rates, home ranges and population density across sexes and seasons. Our results unravel a movement system characterised by many highly sedentary individuals and few relatively mobile ones (many stayers, few movers).

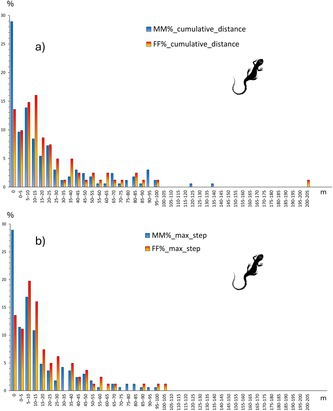

## 1 Introduction

Understanding how animals move through their environment is central to ecology and conservation. Movement determines access to resources and mates, exposure to threats and ultimately population persistence. A key spatial concept is the home range, that is, the area in which an individual performs its regular activities (Burt 1943). Quantifying home range provides spatially explicit information for conservation planning, particularly in fragmented landscapes (Powell and Mitchell 2012; Kays et al. 2015). These spatial metrics are also critical for conservation, as they help identify key habitat requirements and anticipate how species with limited dispersal capacity may respond to environmental change and habitat fragmentation (Kays et al. 2015; Luedtke et al. 2023).

Amphibians are among the most threatened vertebrates worldwide (Stuart et al. 2004; Luedtke et al. 2023), as they face increasing threats from habitat destruction, climate change, pollution, diseases and invasive species (Luedtke et al. 2023). Given their strong dependence on fine‐scale moisture and habitat connectivity, studies of movement ecology are particularly informative for assessing vulnerability to microhabitat alteration and fragmentation (Beebee and Griffiths 2005; Wells 2007; Rittenhouse and Semlitsch 2007). Amphibian space use is generally limited but highly variable across taxa, life stages, reproductive strategies, environmental gradients, intraspecific interactions, predation risk and seasons (Wells 2007; Madison 1997). In urodeles (i.e., tailed amphibians, including newts and salamanders), movements are often particularly localised, with strong site fidelity and small home ranges, although occasional long‐distance displacements can occur (Degani 1978; Bonato and Fracasso 2003; Wells 2007). In many amphibians, spatial behaviour also varies seasonally in relation to reproductive activity, with individuals often concentrating near breeding sites during the reproductive period and dispersing into terrestrial habitats outside of it (e.g., Zamudio and Wieczorek 2006; Wells 2007; Davis et al. 2023). For threatened species in particular, such as *Salamandrina perspicillata* , the lack of quantitative information on space use and movement represents a critical knowledge gap. Fine‐scale spatial metrics are essential to identify key habitat requirements, assess functional connectivity and inform management actions aimed at mitigating habitat fragmentation and environmental change.

Due to their localised occurrence, movement patterns of amphibians often need to be estimated together with population density. Estimating amphibian density is challenging because detection of individuals is imperfect and heterogeneous in space and time (Griffiths et al. 2015; Storfer 2003). Spatially explicit capture‐recapture (SCR) models integrate encounter histories with capture locations to estimate density while simultaneously inferring the spatial scale of movement (Efford 2004; Royle et al. 2014). SCR is increasingly used in herpetology, including in studies on urodeles (Sutherland et al. 2016; Rosa et al. 2025). Combining SCR and a simple, geometry‐based estimate of the minimum area, with minimal assumptions (MEC: minimum enclosing circle; Welzl 1991), offers complementary perspectives. MEC describes realised individual space use, whereas SCR provides detection‐corrected, population‐level movement and density estimates. Additionally, in amphibians, spatial behaviour is frequently shaped by sex‐specific ecological and reproductive strategies, as males and females may differ in movement patterns, habitat use and spatial requirements, especially during breeding periods (Wells 2007; Dodd Jr. 2010). Despite the potential effects of reproductive behaviour and habitat use on sex differences (Wells 2007; Madison 1997), integrative approaches explicitly addressing sex‐specific spatial patterns remain rare in amphibian studies.

Regardless of the recognised importance of fine‐scale spatial data for amphibian conservation, it remains unclear how space use varies among sexes and seasons. Such variation is particularly important in forest‐dwelling urodeles, where reproductive behaviour, microhabitat requirements and seasonal activity patterns can generate strong spatial heterogeneity. In particular, if space use differs between sexes or concentrates near breeding habitats during specific periods, conservation strategies should explicitly account for these dynamics to effectively protect populations.

In this study, we address these knowledge gaps by quantifying fine‐scale spatial ecology in the forest specialist *Salamandrina perspicillata*. By quantifying movement distances, estimating home range sizes through MEC and applying spatial capture‐recapture (SCR) models, we provide an integrated assessment of individual and population space use. Although the terrestrial ecology of *S. perspicillata* has been extensively investigated in recent years (Angelini et al. 2007; Bruni and Romano 2011; Salvidio et al. 2012; Romano et al. 2018), particularly in relation to habitat requirements in forest ecosystems (Basile et al. 2017; Piraccini et al. 2017; Romano et al. 2017; Costa et al. 2022), key aspects of its spatial ecology remain unknown and quantitative estimates of individual space use and population‐level movement parameters are still lacking for the entire *Salamandrina* genus. Specifically, we (i) quantified individual displacement (maximum distance moved) and space‐use extent, (ii) tested whether space use differed between sexes and seasons, and (iii) used multi‐session SCR to estimate sex‐ and season‐specific movement scale (σ) and density patterns in relation to distance from the breeding stream. Based on the species' reproductive ecology, we expected movement activity and space use to vary seasonally, with increased displacement during post‐reproductive periods. We also expected sex‐specific differences in spatial behaviour, reflecting distinct reproductive roles and habitat use. Finally, we expected density to concentrate closer to the stream during spring, when reproduction occurs and to become more spatially spread in autumn.

## 2 Materials and Methods

### 2.1 Study Species

*Salamandrina perspicillata* (Savi, 1821), the Northern spectacled salamander, is a small, forest‐dwelling salamander endemic to northern and central Italy (Romano, Mattoccia, et al. 2009; Romano and Costa 2025). Recently, it has been classified as Endangered by the IUCN, following the inclusion of the threat posed by the fungal pathogen *Batrachochytrium salamandrivorans* in extinction‐risk models. This salamander has a biphasic life cycle: adults are primarily terrestrial, occupying shaded and humid microhabitats in temperate woodlands and, locally, Mediterranean habitats, but females enter water briefly in spring solely to spawn (Lanza 1983; Angelini et al. 2007). Terrestrial activity is quasi‐bimodal, with peaks in spring and autumn (Romano and Costa 2025). Sexual maturity in female *Salamandrina* occurs at 4 years in about 80% of individuals and at 5 years in the remaining 20% (Bovero et al. 2006; Angelini et al. 2010). The maximum recorded lifespan is 12 years, although this value is known only for females. Because our study included only sexually mature individuals (i.e., sexed adults), their potential remaining lifespan would be at most 7–8 years. Therefore, the duration of our study (one and a half years) represents a meaningful portion of the adult lifespan, corresponding to roughly 20% of the potential adult life based on maximum lifespan estimates.

### 2.2 Study Area and Data Collection

**FIGURE 1:**
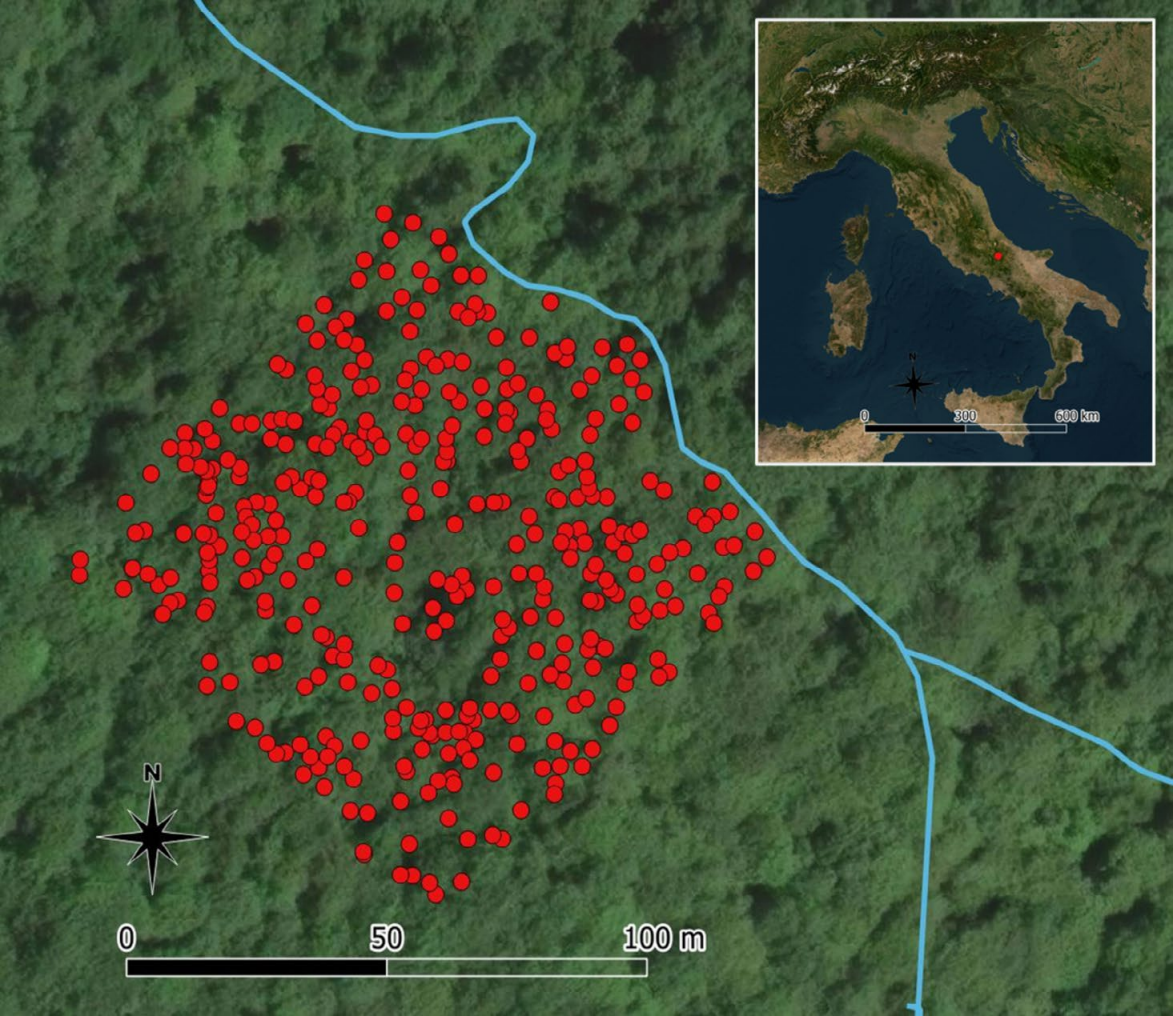
Study area and spatial sampling design within the Collemeluccio Montedimezzo UNESCO Biosphere Reserve (Central Italy). The map shows the first‐order stream used as breeding habitat by *Salamandrina perspicillata* and the spatial distribution of all georeferenced trees within the study plot (red dots). Trees were treated as sampling sites and functioned as spatial detectors in spatial capture‐recapture analyses. The study area covers approximately 1 ha of deciduous forest. Sampling was conducted on one side of the stream due to ecological and methodological considerations related to habitat structure and species ecology (see Study Area section for details).

The study was conducted in a temperate deciduous forest within the Collemeluccio‐Montedimezzo UNESCO Biosphere Reserve (Molise, Central Italy; 41.76232° N, 14.21856° E), crossed by a first‐order stream used by *Salamandrina perspicillata* for reproduction. All trees with a diameter at breast height (DBH) ≥ 10 cm (*n* = 388) within a 1‐ha square plot (Figure 1) were georeferenced with centimetric precision using Field‐Map technology (IFER Ltd., Czech Republic). In SCR modelling, these trees were considered as spatial detectors analogous to sampling traps, due to the salamander's habit of seeking shelter inside tree root holes and underneath tree buttresses (Piraccini et al. 2017). Therefore, each tree was used as a capture location during the field sampling and for modelling individual movements and population density. The sampling design area was restricted to one side of the stream. Including the stream within the sampling grid would have resulted in partitioning the terrestrial habitat and reducing the ability to detect spatial patterns relative to distance from the breeding site. In addition, such design could have introduced bias due to sex‐specific habitat use, as males are predominantly terrestrial whereas females enter the stream only during reproduction (Lanza 1983; Angelini et al. 2007). Moreover, the opposite side of the stream was not included due to its more heterogeneous habitat structure and lower suitability, as preliminary surveys indicated a lower occurrence of salamanders.

Fieldwork was conducted during optimal weather conditions for salamander activity, that is, light rain or drizzle, no wind and ambient temperatures above 8°C, following established protocols (Vanni 1980; Angelini et al. 2007). Surveys were performed by teams of 4–7 trained observers, each session beginning ~2 h after sunrise and lasting ~3 h. During each survey, the area around each tree (within a ~1.5 m radius, see Piraccini et al. 2017 for further details) was carefully inspected by gently removing and replacing the leaf litter, examining the base and root structures, and using flashlights to detect salamanders in crevices and holes. Sex was determined in the field using the cloacal feature as shown by Romano, Bruni et al. (2009) and Vignoli et al. (2010).

We analysed spatial movement and home range patterns of adult individuals captured during a multi‐session capture‐mark‐recapture study. Capture sessions were organised across three seasonal periods: first autumn (session 'autumn 2013ˈ, 08–29 October 2013; four sampling occasions), spring (session sapring', 09 April—31 May 2014; six sampling occasions) and second autumn (session 'autumn 2014ˈ, 03 October—07 November 2014; eight sampling occasions). Each salamander was individually identified from a digital photograph of its ventral pattern, which is unique to individuals and persistent (e.g., Vanni et al. 1997; Angelini et al. 2010). The software Aphis was used for automatic pattern recognition (Moya et al. 2015). Only individuals with confirmed adult status and sex (M = male, F = female), and spatial coordinates, were included in the analyses. As a measure of each salamander's size, we used the snout‐vent length (SVL, in mm) recorded at the first capture.

To investigate whether interannual differences in environmental conditions could have contributed to the observed variation in movement patterns, we explored meteorological data corresponding to the study periods. Given that the dataset includes two autumn sessions (Autumn 2013 and Autumn 2014) but only one spring session, the analysis focused on autumn conditions, allowing a more robust comparison of interannual variability while avoiding confounding effects due to unbalanced seasonal replication. Meteorological data were obtained from an online historical archive (3BMeteo: https://www.3bmeteo.com/) for the nearest available station located in San Pietro Avellana (Isernia), approximately 3.5 km from the study area. We extracted daily data for precipitation (mm), minimum temperature (*T*_min_, °C) and maximum temperature (*T*_max_, °C) corresponding to the sampling periods of the study, first autumn (2013) and second autumn (2014). Mean daily temperature (*T*_mean_) was calculated as the average of *T*_min_ and *T*_max_. To characterise temporal patterns in environmental conditions, we computed monthly and seasonal (September–November) summary statistics, including mean values, standard deviation, and range (maximum‐minimum) for each variable. Differences between years (autumn 2013 vs. autumn 2014) were assessed using Welch's *t*‐tests for mean comparisons and Levene's tests for homogeneity of variances. In addition, to explore the occurrence of potentially relevant environmental triggers for salamander movement, we quantified the frequency of extreme conditions using percentile‐based thresholds calculated over the combined dataset (2013–2014). Specifically, we identified days with high precipitation (above the 90th percentile), warm days (*T*_max_ above the 90th percentile), and cold days (*T*_min_ below the 10th percentile). These indices were used to compare environmental conditions between sampling periods and to support the ecological interpretation of movement patterns.

### 2.3 Spatial Analyses

#### 2.3.1 Individual Movement Dynamics and Spatial Displacement

To characterise individual movement, two questions were addressed: (Q1) how frequently individuals moved and (Q2) how far individuals displaced over the study period. To quantify movement rate (Q1), we estimated individual movement rate (MR) based on successive capture locations. For each individual captured at least twice, we calculated the Euclidean distance (*D*_max_, in metres) between two consecutive captures, and divided it by the minimum number of days elapsed between those captures (Days), standardising the value on an annual basis as: MR = (*D*_max_/Days) × 365. Movement rate was, therefore, expressed in metres per year (m/year), allowing direct comparison among recapture intervals of different duration. This measure has been widely used as a proxy for spatial displacement (Wilson and Anderson 1985; Hammond and Anthony 2006; Tobler and Powell 2013). For each individual, only the maximum movement rate value recorded during the entire study period was retained, in order to avoid pseudo‐replication and to focus on the strongest displacement events. Because the time interval between consecutive captures differed markedly among sampling sessions (1st autumn ≈ 265 days; spring ≈ 90 days; 2nd autumn ≈ 9 days), movement rate was expected to be affected by sampling bias related to recapture interval length. To account for this effect, movement rate was modelled as a function of the number of days between captures using linear regression, and the resulting residuals were used as an index of movement intensity that was independent of recapture interval length. All subsequent comparisons among sessions and between sexes were based on these residual values. The use of residuals represents a pragmatic solution to correct for unequal recapture intervals, given the exploratory nature of movement‐rate analyses. Differences in movement rate among sessions and between sexes were tested using non‐parametric tests due to strong deviations from normality (Shapiro Wilk test: *W* = 0.43, *p* < 0.001). Pairwise comparisons were performed using Mann–Whitney *U* tests with Benjamini‐Hochberg correction for multiple testing (α = 0.05). To assess whether the use of maximum movement rate per individual could introduce bias related to the number of capture opportunities, we compared MR values among individuals with different numbers of capture events using Kruskall–Wallis and Mann–Whitney *U* tests.

To quantify spatial displacement over the entire study period (Q2), we calculated distance‐based movement metrics. Capture locations of each individual were ordered chronologically, and Euclidean distances between successive recaptures were computed. Cumulative movement distance was calculated as the sum of all step lengths and represented a minimum estimate of total spatial displacement over the study period. In addition, we calculated the maximum step length, defined as the largest recorded distance between two consecutive captures of the same individual. These metrics were summarised using distance classes, distinguishing individuals showing no detectable movement, that is, when they remained within a 1.5 m radius of the detector (reported as 0 m for convention), from those exhibiting positive displacement, which were grouped into 5‐m intervals. Indeed, given the spatial resolution of the sampling design, small‐scale movements occurring within the same detector area could not be resolved. Frequencies and percentages were calculated for all individuals and separately for males and females, allowing inter‐individual heterogeneity and sex‐specific patterns in spatial displacement to be evaluated.

Finally, to assess whether individual movement was related to body size, we tested the relationship between snout‐vent length (SVL) and both cumulative movement distance and maximum step length. Analyses were conducted separately for males and females, and were performed including all individuals (i.e., including zero‐displacement cases) and considering only individuals showing positive displacement. Pearson's correlation coefficient was used to test linear relationships, while Spearman's rank correlation was applied to account for non‐normal movement distributions. These statistical analyses were performed using the software PAST version 4 (Hammer et al. 2001).

#### 2.3.2 Home Range Analysis

To estimate home range size in *Salamandrina perspicillata* , we applied the Minimum Enclosing Circle (MEC) method, a geometric technique that identifies the smallest possible circle encompassing all capture locations of an individual. This method does not require smoothing parameters or probabilistic assumptions and is well‐suited for datasets with limited spatial points (Calenge 2006). We implemented Welzl's algorithm (Welzl 1991), an efficient recursive approach operating in expected linear time, to calculate the MEC for each individual with ≥ 2 capture coordinates. We retained all MECs, including those derived from individuals with closely spaced locations, to avoid excluding low‐mobility salamanders. This approach has been successfully applied in spatial ecological studies where traditional kernel‐based methods may underperform due to sparse or clustered data. Furthermore, kernel‐based methods are not accurate estimators of home‐range size, particularly for herpetofauna (Row and Blouin‐Demers 2006). Notably, similar techniques have been used in studies on other small‐bodied forest species, including amphibians and birds, for which irregular or low‐resolution spatial data are common (Johnston and Frid 2002; Blomquist and Hunter Jr. 2009; Muñoz et al. 2016; Oliveira et al. 2023).

A key issue in this analysis concerned the treatment of individuals whose home range values were calculated as zero. These cases occurred when all recorded recapture points for a given individual had identical coordinates to the capture point, indicating no detectable movement (HR = 0) and high site fidelity. MEC values equal to zero should be interpreted as absence of detectable displacement among capture locations rather than complete absence of movement, because small‐scale movements within the search area associated with the same detector could not be detected. Therefore, we addressed two other questions: (Q3) whether home range sizes differed between sexes for all individuals, including sedentary individuals (HR = 0), and (Q4) whether home range sizes differed between sexes among mobile individuals only (HR > 0). Statistical comparisons of home range sizes between males and females were performed using the Mann–Whitney *U* test, a non‐parametric alternative that does not assume normality or homogeneity of variance. This choice was based on exploratory analyses showing strong violations of parametric assumptions (Shapiro–Wilk tests revealed non‐normal distributions in both sexes, *p* < 0.001; Levene's test indicated significant heteroscedasticity, *p* < 0.0001; visual inspection confirmed heavy right‐skewed distributions and a high proportion of zero values). Analyses were performed with the software PAST ver.4 (Hammer et al. 2001).

To evaluate whether body size influenced spatial behaviour, we tested for correlations between SVL and maximum movement distance, and between SVL and home range area (MEC). Separate analyses were conducted for males and females. Due to the non‐normal distribution of spatial variables, Spearman's rank correlation was used (Zar 1999). We did not apply mixed‐effects models due to the strong deviation from normality, zero inflation and limited repeated measures per individual, which could compromise model assumptions and stability. We additionally tested for potential correlations between the average diameter at breast height (DBH) of trees within the MEC home range of each individual and the home range radius through a Spearman's rank correlation test.

#### 2.3.3 Spatially Explicit Capture‐Recapture

We estimated salamander density with multi‐session spatial capture‐recapture models (SCR) with package *secr* (M. Efford 2024) in R, version 4.4.2 (R Core Team 2024), within the RStudio environment (Posit Team 2024). In the SCR framework, a spatially‐explicit state space is used to estimate the location of individuals' activity centres and their detection probability, which is assumed to decrease as distance from the activity centre increases, following the shape of a detection function. The decline of the detection probability with distance is controlled by the 𝝈 parameter, which is related to the home range size of the individuals. We created the state space with a buffer of 50 m (equivalent to roughly four times the mean of the observed maximum distances) around the study area, and with a spatial resolution of 5 m between state space points. We checked the adequacy of the buffer width through the function *esaPlot* within the *secr* package. For each state space point, we calculated the linear distance to the stream in the software QGIS (QGIS Development Team 2024). We modelled males and females separately, and modelled density (*D*) as a function of the interaction between session and distance to the stream. Hence, our model assumed a variation in density with the distance from the stream depending on the session, due to the reproductive phenology of *Salamandrina perspicillata* , with higher densities close to the water during the spring mating season (Basile et al. 2017). Given potential differences in movement and space use between females and males, we hypothesised a different 𝝈 between sexes and different baseline detection probabilities *g*0 among sessions and sexes. The SCR models had, therefore, the following structure for both males and females:

$$\log\left(D\right)\sim \text{dist}\_\text{stream}\times \;\text{session}$$

$$\text{logit}\left(g0\right)\sim \text{session}$$

log(σ)~session

We did not use variable selection, as it can bias inference (Whittingham et al. 2006), but rather we interpreted results based on the coefficient estimates and their confidence intervals. We used AICc (Akaike Information Criterion corrected for small sample size) to compare the half‐normal and the negative exponential detection functions, retaining the function that led to the lower AICc (M. Efford 2024). From the global model with the best detection function, we extracted spatial predictions on the density of salamanders' activity centres through the function *fxTotal* and the predicted density surface with the *predictDsurface* function.

## 3 Results

### 3.1 Individual Movement Dynamics and Spatial Displacement

We recorded 607 detections of 384 individuals, of which 227 (59%; 76 females and 151 males) were captured at least twice. The median of the maximum straight‐line distance between capture points of female individuals was 15.8 m (range = 1.2–138 m; Figure S1), whereas for male individuals was 13.2 m (range = 0.5–115 m; Figure S1); the difference between sexes was not statistically significant.

**FIGURE 2:**
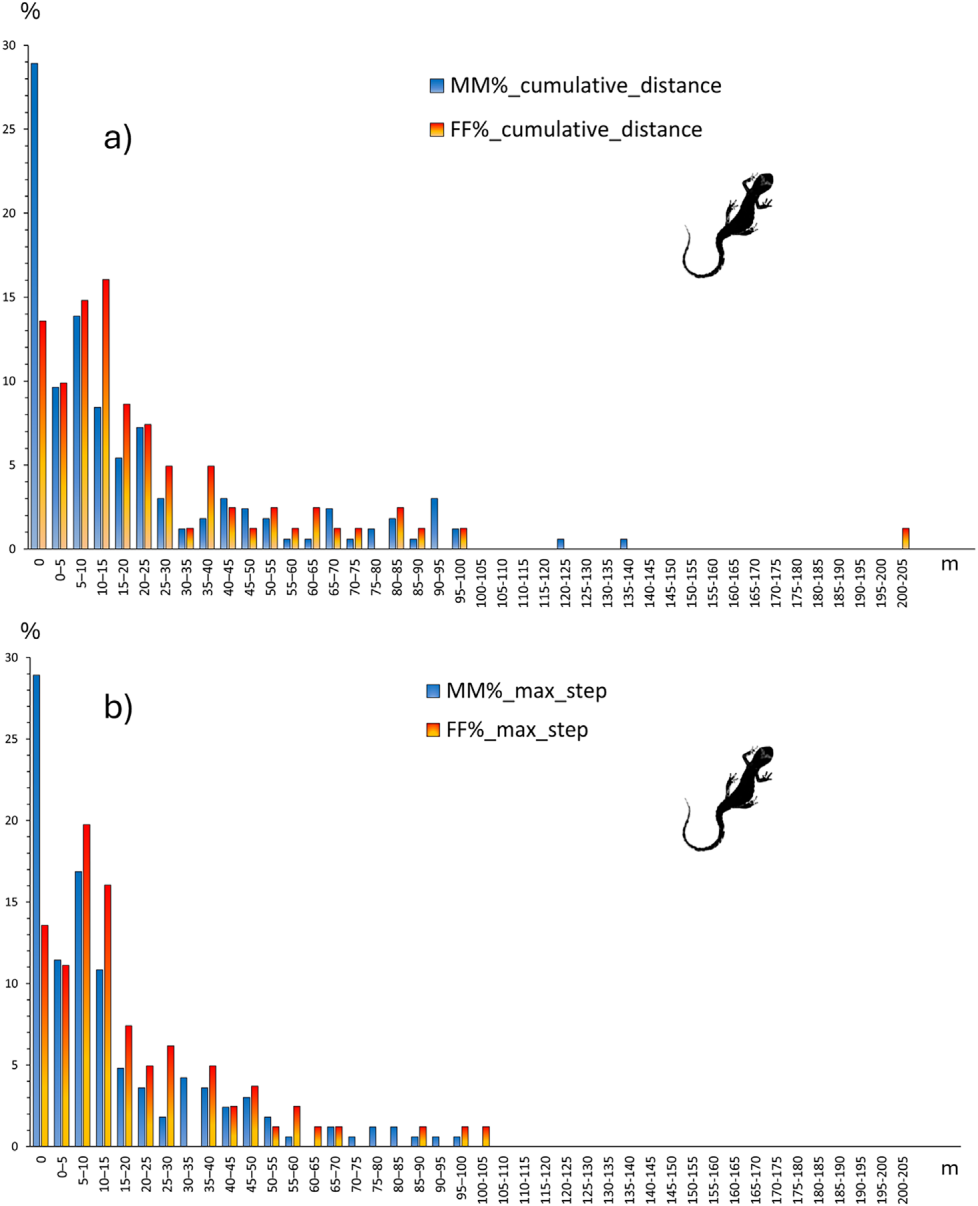
Sex‐specific distributions of individual movement metrics in adult *Salamandrina perspicillata* . For each sex, cumulative movement distance and maximum step length between two successive recaptures are shown separately for males (a) and females (b). Bars represent the percentage of individuals per distance class, with a distinct class for individuals showing no detected movement (0 m) and subsequent classes grouped into 5‐m intervals (> 0–5 m, > 5–10 m, etc.). Both metrics show strongly right‐skewed distributions in both sexes, with most individuals exhibiting no or very limited movement and a small proportion contributing to the right tails. Males display a slightly broader distribution of maximum step length, whereas cumulative movement distances show a comparable or slightly broader spread in females, indicating sex‐specific differences in the expression of inter‐individual variability rather than consistent differences in overall movement extent.

Movement rate analyses revealed marked temporal heterogeneity in individual activity (Q1). The Kruskal–Wallis test to assess bias in MR related to the number of capture opportunities detected overall differences among groups (*H* = 28.9, *p* < 0.001). Pairwise comparisons showed that individuals captured only twice tended to display lower MR values compared to those captured three and five times (*U* = 2445, *p* = 0.001; *U* = 284, *p* = 0.005), but not with those captured four times (*U* = 801, *p* = 0.12). However, groups with higher numbers of capture events were represented by very few individuals (*N* ≤ 18), and MR distributions were highly right‐skewed (Table S1), indicating that these differences were largely driven by a small number of extreme values rather than a systematic effect of recapture frequency. Movement rate varied significantly across sampling sessions, with residual values peaking during the first autumn session (autumn 2013), whereas spring and second autumn (autumn 2014) sessions were characterised by lower and more homogeneous movement rates. This pattern indicates seasonally variable movement rate, with periods of heightened activity interspersed with more stationary. This temporal heterogeneity was confirmed by formal comparisons of residual movement rates among sampling sessions and between sexes. Pairwise comparisons revealed significant differences in residual movement rate among sampling sessions, with values being significantly higher during autumn 2013 compared to both spring and autumn 2014 (*p* < 0.001 in both comparisons). No significant difference was detected between spring and autumn 2014 session (*p* = 0.24; Figure 2). A significant difference between sexes was also detected, with females showing higher residual movement rates than males (*U* = 6902, *p* = 0.013). Overall, the highest residual movement rates were consistently observed during the autumn 2013 session, whereas lower and more comparable values characterised spring and autumn 2014.

Distance‐based movement metrics revealed a strongly asymmetric structure that was not captured by movement rate alone (Q2). Both cumulative movement distance and maximum step length showed strongly right‐skewed distributions (Figure 2). A substantial proportion of individuals fell into the zero‐displacement class, indicating no detectable spatial displacement between detector locations across successive recaptures. Among individuals showing movement, most accumulated very short distances, with the highest frequencies concentrated in the 0–5 m class, followed by a rapid decline in higher distance classes. Only a small number of individuals exhibited larger cumulative distances, generating a long but sparse right tail in the distribution, and only one individual exceeded 200 m of cumulative displacement. Maximum step length was even more strongly concentrated in the lowest distance classes, indicating that large single displacement events were rare (Figure 2). Sex‐specific analyses revealed broadly similar spatial displacement patterns in males and females. Males showed a slightly broader distribution of both cumulative movement distance and maximum step length, with a higher proportion of individuals occupying the upper distance classes, whereas females were more strongly concentrated in the lowest classes; however, in both sexes the majority of individuals remained highly localised. Formal comparisons indicated that cumulative movement distance did not differ significantly between sexes, either when including all individuals or when analyses were restricted to individuals exhibiting positive displacement. For maximum step length, a significant sex difference emerged only when zero‐displacement class individuals were retained in the analysis, reflecting that the effect was driven by differences in the proportion of non‐moving individuals rather than by differences in displacement magnitude among mobile individuals.

Body size (SVL) was not significantly correlated with either cumulative movement distance or maximum step length in either sex. In males, Spearman's rank correlations were weak and non‐significant for both cumulative distance (ρ = −0.01, *p* = 0.92 including zero‐displacement class individuals; ρ = −0.03, *p* = 0.74 excluding zero‐displacement class individuals) and maximum step length (ρ = −0.03, *p* = 0.67 including zeros; ρ = −0.08, *p* = 0.37 excluding zeros). Similarly, in females, no significant relationships were detected between SVL and cumulative movement distance (ρ = 0.11, *p* = 0.32 including zeros; ρ = 0.14, *p* = 0.25 excluding zeros) or maximum step length (ρ = 0.11, *p* = 0.34 including zeros; ρ = 0.14, *p* = 0.26 excluding zeros).

### 3.2 Home Range Estimation

Home range (HR) estimates based on Minimum Enclosing Circles (MEC) varied markedly across individuals and between sexes. When all individuals were considered (Q3), including those with HR = 0, home range size varied widely in both sexes. Females exhibited a higher mean (*N* = 76; 818.82 ± SD 1623.70 m^2^) and median HR (122.92 m^2^) than males (*N* = 151; mean = 663.05 SD ± 1411.76 m^2^; median = 63.62 m^2^) and the difference between sexes was statistically significant (*U* = 4792, *p* = 0.042; range 0–8451 m^2^ for females and 0–7453 m^2^ for males). When individuals with HR = 0 were excluded (Q4), median HR was larger in males (160.22 m^2^) than in females (145.30 m^2^), but the mean, conversely, was slightly smaller in males (848.48 ± SD 1548.07 m^2^ vs. 889.00 ± 1674.02 m^2^), although this difference was not statistically significant (*U* = 3985, *p* = 0.6877). This subset included 70 females and 118 males, with HR values ranging from 0.79 to 8450.88 m^2^ for females and from 1.57 to 7453.43 m^2^ for males. In both analyses, the distributions of HRs were highly skewed and exhibited long upper tails (Figure S2). This is also reflected in the discrepancy between the means and medians in both sexes. For instance, among all females, the mean HR was 818.82 m^2^, while the median was only 122.92 m^2^, indicating that a few individuals with very large HRs heavily influenced the average.

No significant correlations were detected between body size (SVL) and either maximum distance moved or home range size in either sex (SVL vs. Home Range Area (MEC): Males: ρ = −0.065, *p* = 0.427; Females: ρ = 0.124, *p* = 0.284). Similarly, no significant correlation was found between Home Range Area (MEC) and mean DBH of trees within the home range in either sex (DBH vs. Home Range Area: Males: ρ = −0.05, *p* = 0.52; Females: ρ = 0.16, *p* = 0.18).

### 3.3 Spatially Explicit Capture Recapture

**FIGURE 3:**
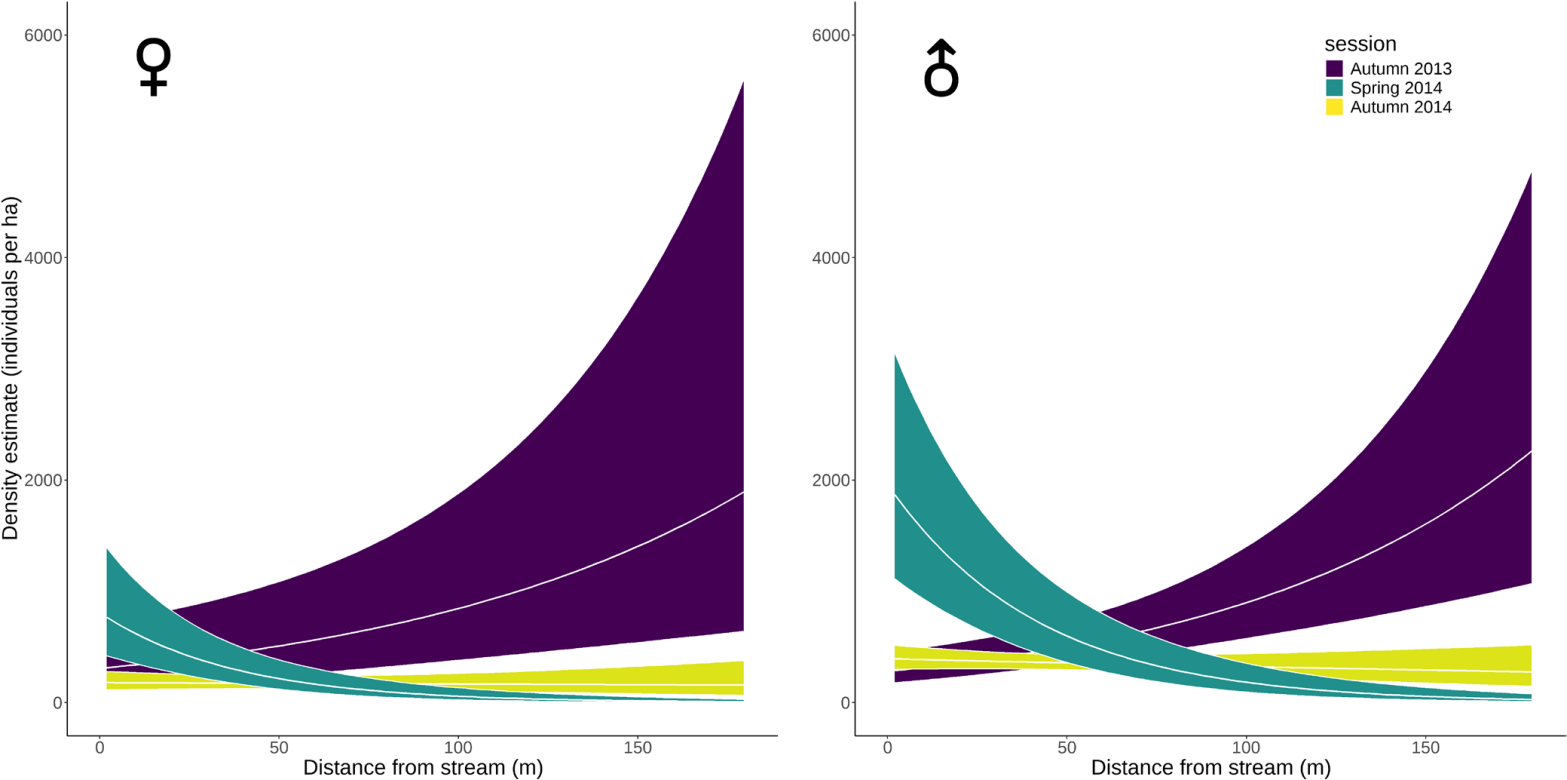
Spatial capture‐recapture model estimates of *Salamandrina perspicillata* females and males' population density in relation to distance from the breeding stream across the three sampling sessions (autumn 2013, spring, autumn 2014). Solid lines represent mean predicted density (individuals per hectare), and shaded ribbons indicate 95% confidence intervals.

**FIGURE 4:**
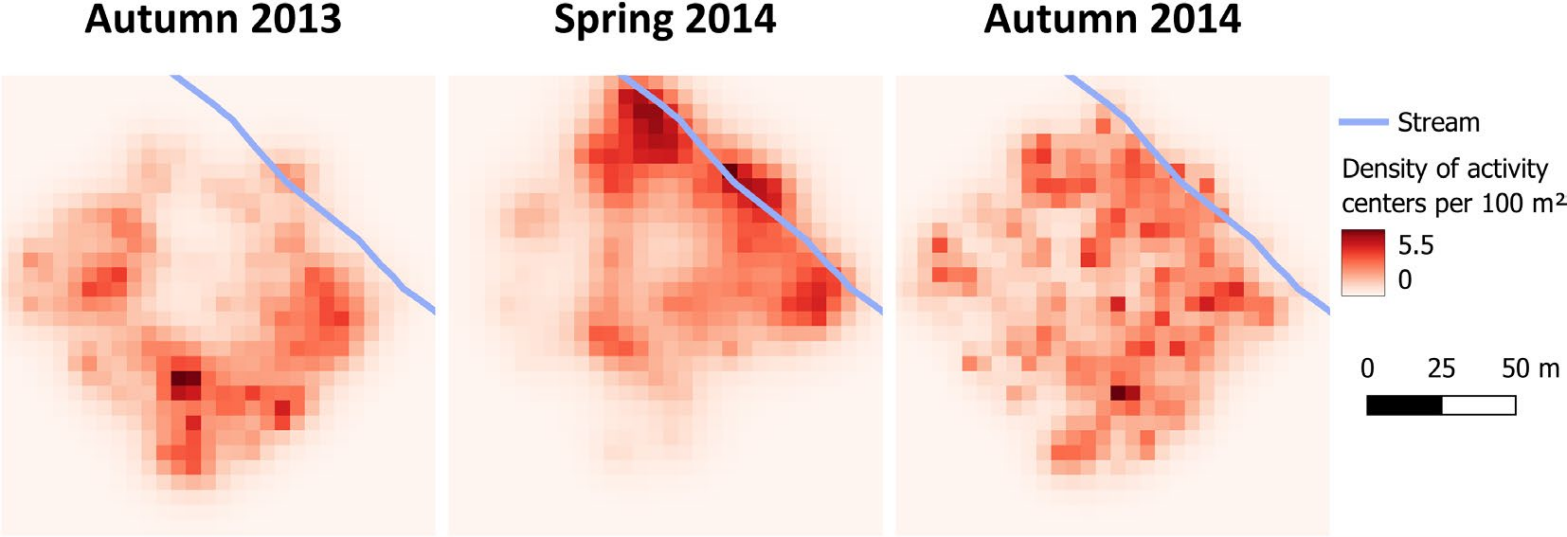
Spatial predictions of activity centre density of males of *Salamandrina perspicillata* derived from spatial capture‐recapture models across the three sampling sessions (autumn 2013, spring, autumn 2014). Colour gradients indicate increasing predicted density of activity centres (per 100 m^2^), while the blue line represents the breeding stream. Seasonal shifts in the spatial distribution of activity centres relative to the stream are evident.

The negative exponential detection function was strongly favoured based on the AICc (ΔAICc = 167.26). The models indicated higher baseline detection probability for males, except for spring (Table 1). The estimated 𝝈 was approximately double for females compared to males during both autumns, while the opposite pattern emerged during spring, where the movement parameter of males was double that of females (Table 1). Density in close proximity to the stream (i.e., *dist_stream* = 0) was markedly higher in spring, especially for males (Females: 807.64 individuals/ha [95% Confidence Interval: 441.09, 1478.83]; Males: 1962.34 individuals/ha [95% C.I. 1161.54, 3315.25]) than in the two autumn sessions (autumn 2013—females: 307.16 [95% C.I. 131.04, 720.00]; autumn 2013—males: 281.72 [95% C.I. 173.70, 456.92]; autumn 2014—females: 179.06 [95% C.I. 113.18, 283.29]; autumn 2014—males: 394.07 [95% C.I. 298.15, 520.86]). However, the variation of density with the distance to the stream was markedly different among sampling sessions: it was higher farther from the stream during the first autumn (Females: *ꞵ‐dist_stream, autumn 2013* = 0.010 [95% C.I. 0.004, 0.017]; Males: *ꞵ‐dist_stream, autumn 2013* = 0.012 [95% C.I. 0.006, 0.017]; Figures 3 and 4), but not in spring, when the opposite pattern emerged (Females: *ꞵ‐dist_stream, spring* = −0.026 [95% C.I. −0.035, −0.017]; Males: *ꞵ‐dist_stream, spring* = −0.024 [95% C.I. −0.030, −0.017]). This is equivalent to a 10% increase in female density every 10 m away from the river in autumn 2013, and a 26% decrease in spring. There was no marked variation in density with the distance from the stream during autumn 2014 (Females: *ꞵ‐dist_stream, autumn 2014* = 0.000 [95% C.I. −0.007, 0.006]; Males: *ꞵ‐dist_stream, autumn 2014* = −0.002 [95% C.I. −0.007, 0.003]). Mean female population density over the whole study area was estimated as 690.47 individuals/ha in autumn 2013, 239.44 in spring, and 170.52 in autumn 2014. Male population density was instead estimated as 728.03 individuals/ha in autumn 2013, 633.05 in spring and 345.83 in autumn 2014.

**TABLE 1:** Summary of SCR models output for females and males in the three sampling sessions. *g0* is the baseline detection probability, while σ is the spatial scale parameter of the detection function.

Sex	Session	Density (ind/ha)	*g*0	σ
Females	Autumn 2013	610.70 [286.38, 1302.33]	0.003 [0.001, 0.010]	8.43 m [4.81, 14.76]
	Spring 2014	135.11 [70.76, 257.99]	0.024 [0.010, 0.056]	3.14 m [2.09, 4.70]
	Autumn 2014	170.42 [119.95, 242.11]	0.005 [0.003, 0.009]	9.97 m [7.45, 13.33]
Males	Autumn 2013	618.84 [420.58, 910.57]	0.019 [0.012, 0.031]	4.55 m [3.93, 5.27]
	Spring 2014	392.86 [227.94, 677.10]	0.005 [0.002, 0.011]	5.99 m [4.30, 8.34]
	Autumn 2014	344.33 [278.37, 425.93]	0.030 [0.021, 0.04]	3.89 m [3.27, 4.64]

*Note:* Density estimates are here reported at the mean distance from the stream (i.e., 67.7 m), as individuals per hectare. Values within square brackets indicate the 95% confidence interval.

### 3.4 Meteorological Results

Meteorological conditions showed limited differences between years during early autumn, but a marked divergence in late autumn (Table S2). In September, precipitation was higher in 2014 compared to 2013 (Table S2), while temperature values (*T*_min_, *T*_max_ and *T*_mean_) were broadly comparable between years, with no significant differences (all *p* > 0.05). October showed similar environmental conditions in both years, with no substantial differences in either precipitation or temperature variables (all *p* > 0.05; Table S2). In contrast, November 2014 was characterised by markedly warmer conditions than November 2013, particularly in terms of *T*_max_ and *T*_mean_ (Table S2). These differences were statistically significant (Welch s *t*‐test: *T*_max_, *p* = 0.001; *T*_mean_, *p* = 0.005), while *T*_min_ showed a marginal difference (*p* ≈ 0.05). Precipitation did not differ significantly between years (*p* > 0.05). Considering the entire autumn period (September–November), no significant differences in mean precipitation were observed between years (*p* > 0.05), although variability was higher in 2014. Temperature patterns, however, revealed a clear interannual signal, with overall warmer conditions in 2014 driven primarily by the anomalies observed in November.

## 4 Discussion

This study aimed at quantifying individual and population‐level space use in the forest‐dwelling salamander *Salamandrina perspicillata* , with a particular focus on sex‐ and season‐specific variation. We expected movement and space use to vary across seasons in relation to reproductive activity, and to differ between sexes due to contrasting ecological roles. By integrating individual‐based and population‐level approaches, we provided a multi‐scale assessment of spatial behaviour of this endemic salamander to Italy.

### 4.1 Sex‐Specific Spatial Behaviour

Our results revealed subtle yet ecologically meaningful sex‐related differences in the spatial ecology of *Salamandrina perspicillata* . Males of *Salamandrina* generally displayed lower inter‐individual variability in both movement distances and home range sizes, whereas females exhibited greater inter‐individual variability in movement distances and larger home ranges. However, the spatial scale parameter (σ) of females was markedly smaller compared to males during spring, in accordance with what is commonly reported for other terrestrial salamanders, where males often display greater mobility during the reproductive period (e.g., Hurlbert 1969; Douglas 1979; Manenti et al. 2017). Conversely, in both autumns, females showed significantly higher σ than males. Considering home‐range size, when analyses were restricted to mobile individuals only, sex differences disappeared. This suggests that observed sex‐related patterns do not reflect a uniform difference in mobility, but rather a broader behavioural spectrum among females, encompassing both zero‐displacement class salamanders and individuals with broader spatial distribution around activity centres.

Although limited spatial recaptures for some individuals may have influenced parameter uncertainty, the concordance between individual‐based (MEC) and population‐level (SCR) approaches strengthens the inference that females tend to use space more variably.

### 4.2 Seasonal Movement Dynamics

Seasonal variation emerged as a major driver of spatial behaviour and population density. Residual movement rates were highest during autumn 2013, while spring and autumn 2014 showed comparably lower values. Using residual movement rate removed the bias related to unequal recapture intervals and also sex differences. In autumn 2013, salamanders exhibited consistently higher movement activity, possibly influenced by more favourable climatic conditions and reflecting seasonal behaviour, such as post‐breeding dispersal or pre‐winter relocation. On the other hand, the higher movement of females compared to males may indicate sex‐specific habitat use and reproductive‐related displacement, given the temporary downstream migration for spawning (Angelini et al. 2007). Also, the movement parameters σ from SCR models indicated an overall wider space use of females compared to males during both autumn sessions, while the opposite pattern emerged in spring, when the movement parameter of females halved. This pattern is likely due to females being closer to the stream in spring for spawning, while males might still move around in search of mating partners. Higher movements of females have already been reported in other European salamanders, such as *Speleomantes strinatii* (Rosa et al. 2025), where females show higher investment in parental care, including the selection of specific microclimatic conditions for hatching or to favour eggs development (Oneto et al. 2014; Lunghi et al. 2015). Thus, both individual‐level and population‐level movement patterns indicate a strong seasonal reorganisation of space use, with implications for both detectability and habitat requirements across the annual cycle.

### 4.3 Influence of Proximity to Aquatic Habitat

Distance from the stream strongly influenced spatial density patterns in a season‐dependent manner. In spring, salamander density was significantly higher near the stream, consistent with its role as a breeding habitat and with the aggregation of individuals in proximity to aquatic microhabitats. In contrast, during autumn 2013, density increased with distance from the stream, suggesting broader dispersion following reproduction, possibly driven by resource exploration or territorial behaviour. These spatial shifts are ecologically meaningful. They support the idea that *S. perspicillata* adjusts its spatial strategy seasonally, a pattern also observed in plethodontid salamanders (Cecala et al. 2014) and in other forest‐dwelling amphibians (Rittenhouse and Semlitsch 2007). No clear spatial gradient was observed during autumn 2014, indicating a return to more homogeneous habitat use. These results highlight the dynamic nature of space use in *S. perspicillata* and emphasise the importance of riparian zones as seasonally critical habitats. Seasonal shifts in the spatial distribution of activity centres underscore the need to account for temporal variability when assessing habitat suitability and population structure in forest‐dwelling amphibians. The lack of a clear spatial gradient during autumn 2014 may also reflect interannual differences in environmental conditions. In particular, autumn 2014 was characterised by warmer conditions compared to autumn 2013, especially during November, suggesting a delayed or less pronounced onset of autumnal cooling (Table S2). Such conditions may have weakened or postponed the environmental cues that typically trigger dispersal away from the breeding stream. As a consequence, autumn 2014 likely included a mixture of individuals still associated with the stream and others already dispersed into terrestrial habitats, resulting in a more homogeneous spatial distribution and the absence of a clear distance‐related pattern. This interpretation is consistent with the strong sensitivity of amphibian movement to fine‐scale climatic conditions, particularly temperature, moisture and hydroperiod. Long‐term studies have shown substantial interannual variability in amphibian abundance, phenology and spatial activity, suggesting that year‐to‐year climatic fluctuations may strongly influence spatial behaviour and potentially obscure simple distance‐based patterns (Peterman and Semlitsch 2014; Dubos et al. 2019). We therefore interpret the differences between autumn sessions as arising from a combination of seasonal and interannual climatic effects, rather than as an inconsistency in the movement pattern.

A potential limitation of this study is the relatively short temporal extent of the dataset (1.5 years), which may not fully capture interannual variability in movement patterns and population dynamics. Year‐to‐year differences in climatic conditions, particularly in temperature and moisture regimes, are known to influence amphibian activity and space use, and may partly explain the variability observed between sampling sessions (e.g., Benard and Greenwald 2023; Dalpasso et al. 2023). However, it is worth noting that the study period represents a substantial proportion of the adult lifespan of *S. perspicillata* (Bovero et al. 2006; see also par. 2.1), providing meaningful insight into individual spatial behaviour. However, differences observed between the autumn 2013 and autumn 2014 sessions may reflect year‐specific environmental conditions rather than purely seasonal dynamics. Additional multi‐year data, including repeated spring sessions, would be necessary to robustly assess the relative contribution of seasonal versus annual effects. Although the limited temporal extent of the study prevents a formal separation of seasonal and year effects, these findings suggest that climatic variability should be explicitly considered when interpreting short‐term movement datasets. Another potential limitation of the present study is the low capture probability of salamanders, which might limit our ability to robustly estimate population density, as suggested by the strong variation in mean density estimates across sessions and their broad confidence intervals. However, very low detection probability is the norm rather than the exception in herpetological studies (Mazerolle et al. 2007). Despite the large confidence intervals and the wide seasonal variation, our density estimates are in line with the mean abundance between 0.48 and 4.02 individuals estimated at 30 m^2^ plots by Rosa et al. (2023). Those estimates would correspond in our study to 4.25, 2.62 and 1.55 individuals per 30 m^2^ for the three sessions, respectively. In order to improve density estimates and their uncertainty, future studies should use a greater number of sampling occasions, to increase recapture probability.

### 4.4 Integrating Individual‐ and Population‐Level Spatial Ecology: Implications for Conservation

Overall, *Salamandrina perspicillata* displayed limited spatial mobility, with typically small home ranges and short movement distances, consistent with patterns reported for many European (e.g., Salvidio and Pastorino 2002; Bonato and Fracasso 2003) and American terrestrial urodeles (Kleeberger and Werner 1982; Ovaska 1988). However, substantial inter‐individual variability suggests that populations comprise a mix of almost sedentary and more mobile individuals, both of which may play distinct ecological roles. Whether highly localised individuals reflect intrinsic behavioural traits or extrinsic habitat conditions remains unclear. Although we tested the relationship between home range size and tree diameter within individual home ranges as a proxy of habitat structure, no significant relationship emerged. However, our data do not allow us to distinguish between these hypotheses, as additional fine‐scale environmental information was not available. Future studies integrating microhabitat and microclimatic variables would be necessary to disentangle these mechanisms.

Our findings highlight the critical importance of fine‐scale habitat continuity for *S. perspicillata* . Its reliance on moist leaf litter, shaded forest interiors and proximity to streambeds for reproduction makes it particularly susceptible to habitat fragmentation and microhabitat alteration (Cushman 2006; Basile et al. 2017). Even small breaks in habitat continuity, such as reductions in canopy cover or litter compaction, could impede movement, limit breeding opportunities, and negatively affect individual survival and population connectivity (Cushman 2006; Rittenhouse and Semlitsch 2007). These effects may be particularly pronounced in species showing seasonal aggregation near aquatic habitats (Piraccini et al. 2017; Marsh et al. 2005), especially in *Salamandarina* whose abundance is negatively affected by increased direct insolation (Rosa et al. 2023). This reinforces the need for forest management strategies that maintain structurally complex and hydrologically stable microhabitats. Our study provides the first quantitative baseline of spatial behaviour for the genus *Salamandrina* and highlights the importance of fine‐scale habitat continuity for the conservation of small forest‐dwelling amphibians. These spatial metrics are essential for designing effective conservation actions and for anticipating how small‐range specialists may respond to environmental change (Walter et al. 2015; Muñoz et al. 2016; Kays et al. 2015; Luedtke et al. 2023). This is particularly relevant, considering the recent implementation of Italy's national framework for the voluntary carbon market (VCM; Ministry of Agriculture, Food Sovereignty and Forestry, October 2025). In Apennine forests, this could promote carbon‐oriented strategies such as the conversion of abandoned coppices into high forests (Nonini and Fiala 2021), as well as preventive actions aimed at limiting rapid CO_2_ release, including fuel reduction through deadwood removal or understory thinning (Hurteau et al. 2008). This emerging scenario underscores the need for forest management guidelines that place biodiversity conservation at their core, ensuring that increasing management intensity for carbon objectives does not compromise habitat suitability for endangered and forest‐specialist species.

By combining individual‐based (MEC and movement metrics) and population‐level (SCR) spatial approaches, our study provides a coherent, multi‐scale perspective on movement strategies in *Salamandrina perspicillata* . MEC estimates capture realised individual space use and reveal strong heterogeneity, with many individuals exhibiting extremely limited or no detectable spatial displacement between detector locations. Movement metrics further demonstrate a strongly asymmetric pattern characterised by “few movers, many stayers” (i.e., relatively few individuals exhibiting large detectable displacements and many highly localised individuals) suggesting that inter‐individual variation in movement plays a central role in shaping population‐level spatial dynamics. However, given the spatial resolution of the detector system (the trees), individuals classified within the zero‐displacement class should not be interpreted as completely immobile, but rather as showing no detectable displacement between detector locations. Because each detector included a search area extending approximately 1.5 m around each tree, small‐scale movements occurring within the same detector area could not be resolved. Therefore, the observed ‘many stayers, few movers’ pattern should be interpreted as reflecting heterogeneity in detectable spatial displacement at the spatial scale of the sampling design. Spatial capture‐recapture models complement these findings by accounting for imperfect detection and linking individual movement behaviour to population‐level movement scale (σ) and density patterns. Together, these approaches illustrate how integrating individual movement descriptors with population‐level spatial models improves inference on movement processes in low‐mobility species. From a conservation perspective, this integrated framework indicates that *S. perspicillata* persistence depends primarily on fine‐scale habitat continuity rather than frequent long‐distance dispersal, highlighting the importance of maintaining microhabitat connectivity in forest ecosystems. Future research should focus on multi‐year datasets with multiple recapture occasions, integration of microclimatic variables and fine‐scale habitat characterisation to better understand the drivers of spatial behaviour and improve conservation strategies.

## Author Contributions

**Antonio Romano:** conceptualization (equal), data curation (equal), formal analysis (equal), investigation (equal), methodology (equal), project administration (equal), supervision (equal), writing – original draft (equal), writing – review and editing (equal). **Andrea Costa:** conceptualization (equal), investigation (equal), methodology (equal), writing – review and editing (equal). **Marco Basile:** conceptualization (equal), investigation (equal), methodology (equal), writing – review and editing (equal). **Giacomo Rosa:** data curation (equal), formal analysis (equal), methodology (equal), writing – review and editing (equal). **Marco Salvatori:** formal analysis (equal), methodology (equal), writing – original draft (equal), writing – review and editing (equal).

## Funding

This work was supported by the European Commission, LIFE09 ENV/IT/000078.

## Conflicts of Interest

The authors declare no conflicts of interest.

## Supporting information


**Figure S1:** Distribution of maximum straight‐line distances between capture locations for male and female *Salamandrina perspicillata*.

**Figure S2:** Distribution of individual home‐range size (Minimum Enclosing Circle, MEC; in m on the *y* axis) for female (in green) and male (in blue) *Salamandrina perspicillata*.

**Table S1:** Descriptive statistics of maximum movement rates of individuals grouped by number of recapture opportunities. CV, coefficient of variation; N_ind, number of individuals for the two sexes; SD, standard deviation; SE, standard error.

**Table S2:** Summary of meteorological conditions during the two autumns studied: first autumn (2013) and second autumn (2014). Values are reported as mean ± standard deviation, with range in parentheses. Seasonal values refer to the September + October + November period (SON).

## Data Availability

The data associated with this manuscript are available in the Supporting Information and will be uploaded to a public repository upon acceptance. The data include the capture history of the *Salamandrina* individuals and the location of the traps, that is, trees.
