# Supplementary material for: Many Stayers, Few Movers: Seasonal and Sex‐Based Movement Patterns in an Endangered Forest‐Dwelling Salamander
Source: Ecol Evol. 2026 Jul 7;16(7):e73900. doi: 10.1002/ece3.73900 (PMC13339926; doi:10.1002/ece3.73900)
Supplement: Supplementary file 1 — Figure S1: Distribution of maximum straight‐line distances between capture locations for male and female Salamandrina perspicillata. [file ECE3-16-e73900-s002.pdf]

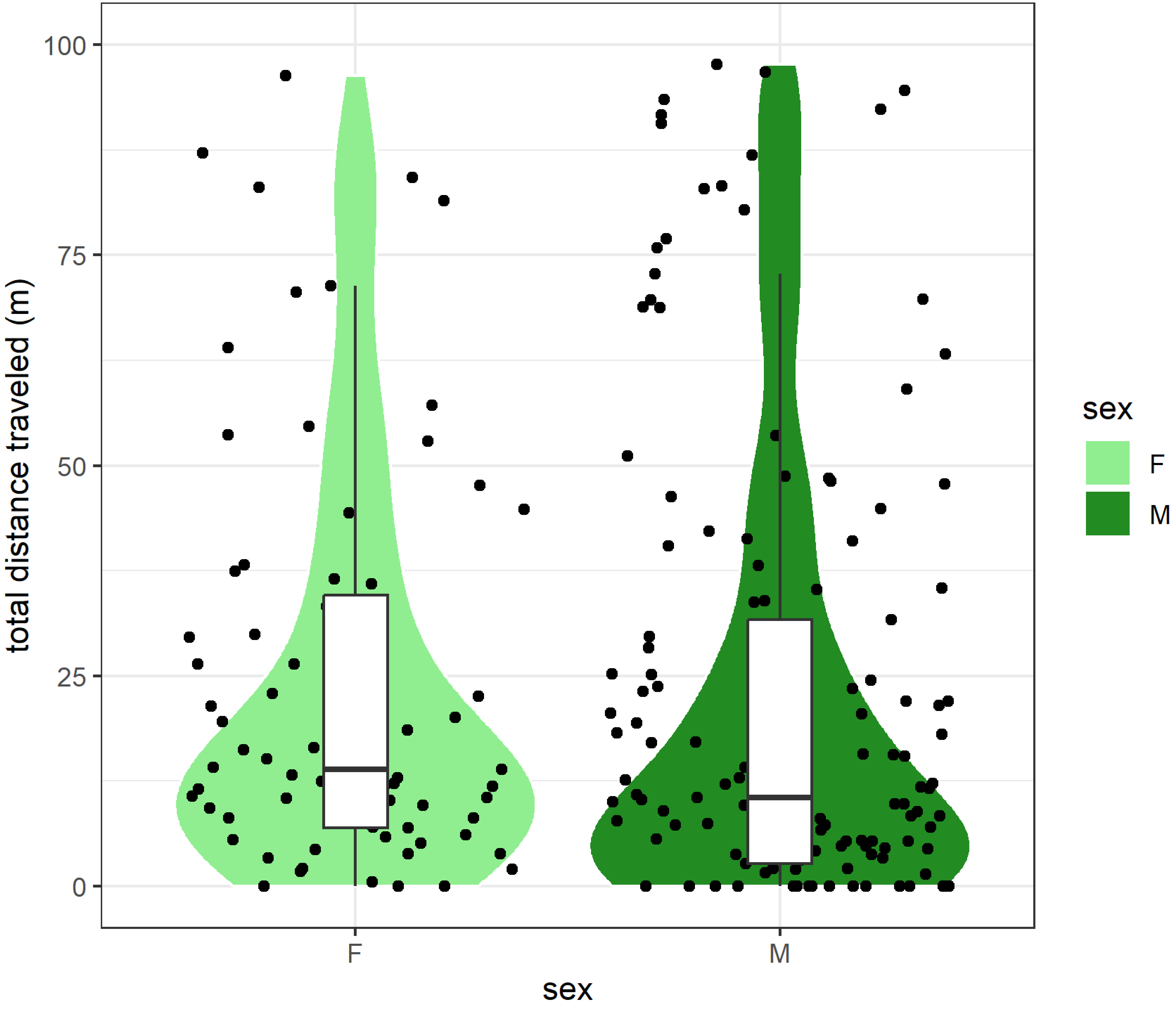

Figure S1. Distribution of maximum straight-line distances between capture locations for male and female *Salamandrina perspicillata*. While females tended to show slightly longer displacement distances than males, the overall variation was high and no significant difference was detected between sexes. The boxplots illustrate the central tendency and spread of movement distances within each group
