# Supplementary material for: Many Stayers, Few Movers: Seasonal and Sex‐Based Movement Patterns in an Endangered Forest‐Dwelling Salamander
Source: Ecol Evol. 2026 Jul 7;16(7):e73900. doi: 10.1002/ece3.73900 (PMC13339926; doi:10.1002/ece3.73900)
Supplement: Supplementary file 2 — Figure S2: Distribution of individual home‐range size (Minimum Enclosing Circle, MEC; in m on the y axis) for female (in green) and male (in blue) Salamandrina perspicillata. [file ECE3-16-e73900-s004.pdf]

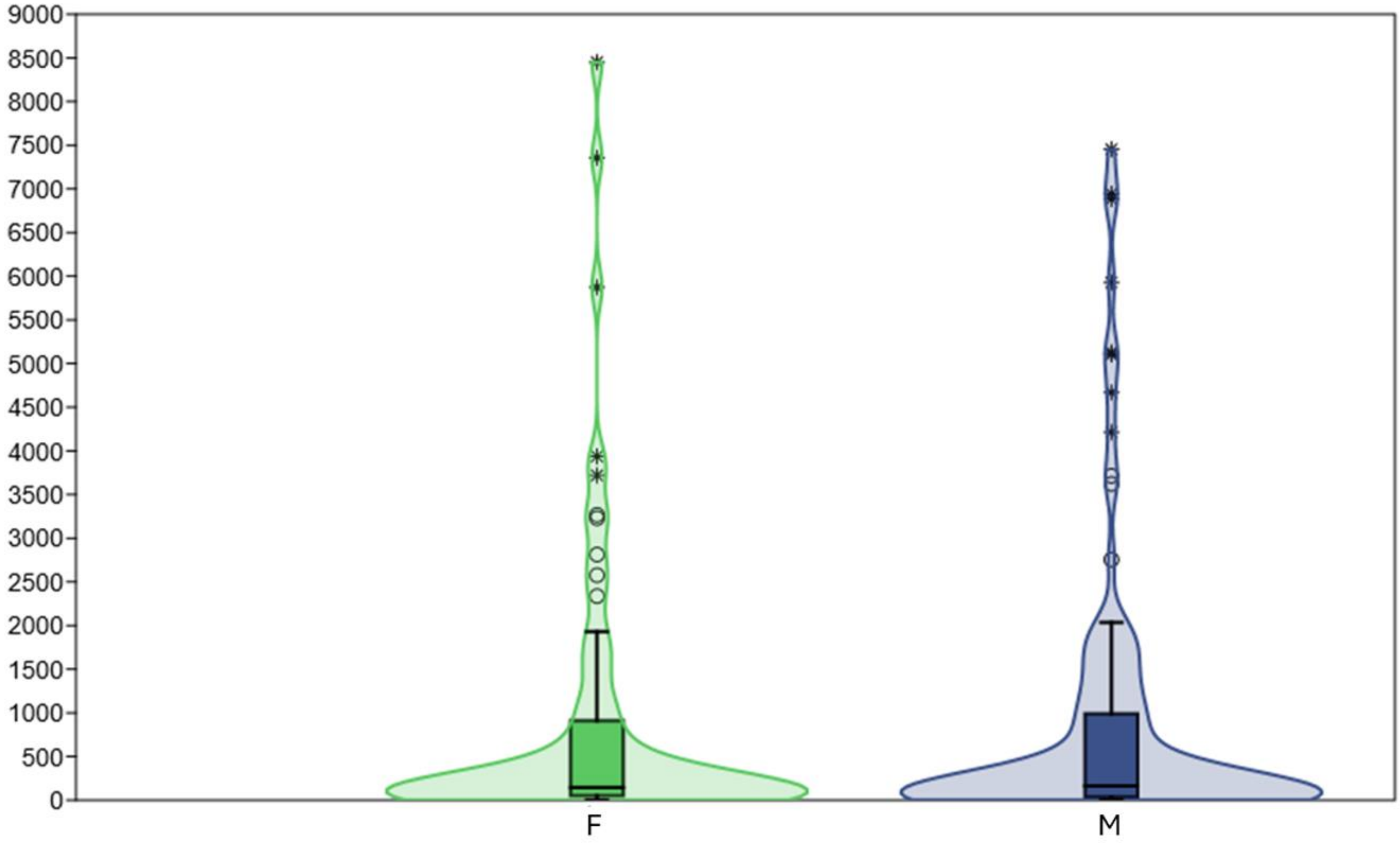

Figure S2. Distribution of individual home-range size (Minimum Enclosing Circle, MEC; in m on the y axis) for female (in green) and male (in blue) *Salamandrina perspicillata*. Violin plots show the full distribution of values, with median (white dot) and interquartile range (thick bars). Home-range size was highly right-skewed in both sexes, with most individuals exhibiting very small spatial extents and a minority showing relatively large values. Zero values represent sedentary individuals with no detected spatial displacement
