# Supplementary material for: Many Stayers, Few Movers: Seasonal and Sex‐Based Movement Patterns in an Endangered Forest‐Dwelling Salamander
Source: Ecol Evol. 2026 Jul 7;16(7):e73900. doi: 10.1002/ece3.73900 (PMC13339926; doi:10.1002/ece3.73900)
Supplement: Supplementary file 3 — Table S1: Descriptive statistics of maximum movement rates of individuals grouped by number of recapture opportunities. CV, coefficient of variation; N_ind, number of individuals for the two sexes; SD, standard deviation; SE, standard error. [file ECE3-16-e73900-s003.docx]

**Table S1.** Descriptive statistics of maximum movement rates of individuals grouped by number of recapture opportunities. N_ind: Number of individuals for the two sexes; SE: Standard Error, SD: Standard Deviation; CV: Coefficient of Variation.

| **Captures** | 2 | 3 | 4 | 5 | 6 |
| --- | --- | --- | --- | --- | --- |
| **N_ind (F; M)** | 140 (50; 90) | 54 (17; 37) | 18 (6; 12) | 11 (1;10) | 2 (1; 1) |
| **Min** | 0 | 0 | 0 | 0.21 | 3.5 |
| **Max** | 76.9 | 81.5 | 17.1 | 203.2 | 4.12 |
| **Mean** | 2.56 | 4.96 | 1.62 | 20.5 | 3.8 |
| **Median** | 0.06 | 0.44 | 0.29 | 0.72 | 3.8 |
| **SE** | 0.77 | 1.79 | 0.95 | 18.3 | 0.32 |
| **SD** | 9.1 | 13.13 | 4.01 | 60.68 | 0.56 |
| **CV** | 355.31 | 264.27 | 248.39 | 296 | 11.98 |
