# Supplementary material for: Many Stayers, Few Movers: Seasonal and Sex‐Based Movement Patterns in an Endangered Forest‐Dwelling Salamander
Source: Ecol Evol. 2026 Jul 7;16(7):e73900. doi: 10.1002/ece3.73900 (PMC13339926; doi:10.1002/ece3.73900)
Supplement: Supplementary file 4 — Table S2: Summary of meteorological conditions during the two autumns studied: first autumn (2013) and second autumn (2014). Values are reported as mean ± standard deviation, with range in parentheses. Seasonal values refer to the September + October + November period (SON). [file ECE3-16-e73900-s001.docx]

**Table S2.** Summary of meteorological conditions during the two autumns studied: first autumn (2013) and second autumn (2014). Values are reported as mean ± standard deviation, with range in parentheses. Seasonal values refer to the September+October+November period (SON).

| **Month** | **Year** | **Precipitation (mm)** | **Tmin (°C)** | **Tmax (°C)** | **Tmean (°C)** |
| --- | --- | --- | --- | --- | --- |
| September | 2013 | 2.20 ± 3.45 (0–15) | 11.01 ± 2.45 (6.2–15.8) | 22.24 ± 3.12 (15.4–27.6) | 16.63 ± 2.60 (10.8–21.5) |
| September | 2014 | 5.37 ± 6.88 (0–32) | 11.45 ± 2.36 (7.1–16.3) | 20.53 ± 2.78 (14.8–25.1) | 15.99 ± 2.48 (10.9–20.7) |
| October | 2013 | 3.06 ± 4.21 (0–18) | 9.97 ± 2.71 (4.8–14.2) | 18.61 ± 2.95 (12.4–23.2) | 14.29 ± 2.63 (9.1–18.5) |
| October | 2014 | 3.10 ± 4.35 (0–20) | 8.55 ± 2.63 (3.9–13.1) | 17.82 ± 2.67 (11.9–22.5) | 13.18 ± 2.39 (8.2–17.8) |
| November | 2013 | 5.94 ± 7.12 (0–30) | 3.87 ± 2.51 (−1.2–8.3) | 9.82 ± 2.84 (3.1–15.6) | 6.84 ± 2.52 (1.5–11.8) |
| November | 2014 | 5.97 ± 7.45 (0–35) | 5.70 ± 2.89 (0.4–11.2) | 13.78 ± 3.47 (6.5–19.2) | 9.74 ± 3.01 (3.6–14.9) |
| **Autumn (SON)** | **2013** | **3.73 ± 6.47 (0–32)** | **8.30 ± 4.40 (−1.2–20.0)** | **16.91 ± 6.68 (3.1–32.3)** | **12.60 ± 5.38 (1.5–26.0)** |
| **Autumn (SON)** | **2014** | **4.79 ± 9.08 (0–58)** | **8.56 ± 3.92 (0.4–16.2)** | **17.38 ± 4.58 (6.5–24.6)** | **12.97 ± 4.05 (3.6–16.2)** |
